# Oxymatrine Inhibits Renal Cell Carcinoma Progression by Suppressing β-Catenin Expression

**DOI:** 10.3389/fphar.2020.00808

**Published:** 2020-06-05

**Authors:** Yinshan Jin, Jiannan Liu, Yadong Liu, Yang Liu, Guiying Guo, Shiliang Yu, Ruihua An

**Affiliations:** Department of Urology, The First Affiliated Hospital of Harbin Medical University, Harbin, China

**Keywords:** oxymatrine, renal cell carcinoma, β-catenin, proliferation, apoptosis, migration, invasion, chemotherapy sensitivity

## Abstract

**Aims:**

Oxymatrine (OMT) has been identified to possess immunomodulatory, antiinflammatory and anticancer properties. This study aimed to investigate its precise function and the underlying molecular mechanisms in renal cell carcinoma progression.

**Methods:**

The antineoplastic effect of oxymatrine was investigated by CCK-8 assay, cell cycle analysis, apoptosis assay, wound healing experiment, transwell assay, and drug-sensitivity analysis in renal cancer cells following oxymatrine treatment. The modulation of oxymatrine on β-catenin was analyzed through western blot and immunofluorescence assay. β-catenin overexpression was employed to determine the key role of β-catenin in oxymatrine-inhibited renal cell carcinoma *in vitro*. In addition, animal model was established to investigate the effect of oxymatrine on tumor growth *in vivo*.

**Results:**

Oxymatrine inhibited renal cell carcinoma progression *in vitro*, including cell proliferation, apoptosis, migration, invasion and chemotherapy sensitivity. Further mechanistic studies demonstrated that oxymatrine exerted its antineoplastic effect through suppressing the expression of β-catenin. Moreover, in nude mice model, oxymatrine exhibited remarkable inhibition of tumor growth, which was consistent with our *in vitro* results.

**Conclusions:**

Our findings illuminate oxymatrine as an effective antitumor agent in renal cell carcinoma, and suggest it a promising therapeutic application in renal cell carcinoma treatment.

## Introduction

Renal cell carcinoma (RCC), one of the most common malignant cancers in urinary system, is with an increased incidence in recent years. This cancer-related death is more than 140,000 per year (Capitanio and Montorsi, 2016; Capitanio et al., 2019). The pathogenesis of RCC is not very clear now, but some potential risk factors have been evaluated to be associated with it, such as obesity, alcohol, smoking, hypertension, urinary stones, diabetes, and liver and chronic kidney diseases (Capitanio et al., 2019). The main treatment for RCC is surgery, including conservative surgery (nephron-sparing surgery) and radical surgery (radical nephrectomy), supplemented with radiotherapy and chemotherapy (Crepel et al., 2010a; Crepel et al., 2010b). Due to the hidden physiological position of the kidney, the early symptoms of RCC are not obvious. Most patients had locally advanced disease at the time of diagnosis, harboring distal or lymphatic metastases, which resulted in high recurrence rate and poor prognosis (Janzen et al., 2003; Ferlay et al., 2015). Therefore, the introduction of effective adjuvant drugs, for postoperative treatment and prevention of recurrence, is considered to improve current therapies for RCC.

Oxymatrine (OMT), a natural quinolizidine alkaloid, is one of the main effective constituents derived from the roots of Chinese herb *Sophora flavescens* (Rabea et al., 2010). A growing body of research illustrates various pharmacological activities of OMT, including antiarrhythmic, antifibrotic, antiviral, antiinflammatory, antiallergic, and cardiovascular protective effects (Deng et al., 2009; Cao et al., 2010; Cui et al., 2010; Chen et al., 2013). Meanwhile, OMT has aroused considerable interest as its antitumor potential in various cancers through diverse signal pathways, such as inhibition of proliferation, induction of apoptosis, suppression of angiogenesis, inhibition of metastasis and enhance the sensitivity of chemotherapy drugs (Guo et al., 2015; Liu et al., 2016; Wu et al., 2017). Nevertheless, little is known about the precise antitumor activity and underlying mechanism of OMT in RCC development.

β-catenin is a founding component of cadherin-based, Ca^2+^-dependent adherens junctions that are highly dynamic (Yap et al., 1997; Valenta et al., 2012). During EMT, a progression activating cancer invasion and formation of metastases (Thiery et al., 2009), reduction of E-cadherin-mediated cell adhesion promotes β-catenin release, accumulation in the cytoplasm and its signal activation (Zeisberg and Neilson, 2009; Heuberger and Birchmeier, 2010). β-catenin not only exerts its structural function in cell-to-cell adhesion, but also plays the key effector of canonical Wnt signaling in the nucleus. In pathological conditions, activation of Wnt signaling results in the disassembly of β-catenin destruction complex and prevention GSK3β-mediated phosphorylation of β-catenin. Under this condition, β-catenin is aberrantly activated and forms complexes with transcription factors, which leads to the progression of various types of cancer (Polakis, 2007; Lucero et al., 2010; Xu et al., 2016). However, the clinical value of β-catenin dysregulation in RCC deserves detailed study.

In this study, we investigated the roles and the underlying mechanism of OMT in RCC. The efficacy of OMT against RCC was evaluated *in vitro*, represented by decreased cell proliferation, metastasis, and increased apoptosis and sensibility of chemotherapeutic drug. In accordance with *in vitro* studies, OMT suppressed tumor progression in mouse models. Furthermore, our results provided the novel mechanism that the antineoplastic function of OMT was dependent on its inhibition of β-catenin in RCC. Overexpression of β-catenin caused completely reverse effects in cell proliferation, apoptosis, and metastasis modulated by OMT. All these findings proved OMT as a potential therapeutic drug for the treatment of RCC.

## Materials and Methods

### Cell Lines and Cell Culture

Human renal cancer cell lines A498 and SW839 were cultured in MEM (Gibco) and RPMI-1640 (hyclone) medium supplemented with 10% fetal bovine serum (BI). All the cells were maintained in incubator at 37°C with 5% CO2.

### Antibodies and Reagents

The primary antibodies recognized as following: CDK6 (Proteintech, 19117-1-AP), p27 (Proteintech, 25614-1-AP), MMP2 (Proteintech, 10373-2-AP), MMP9 (Proteintech, 10375-2-AP), Histone H3 (Proteintech, 17168-1-AP), β-actin (Proteintech, 60008-1-Ig), GSK3β (Bioss, bs-0023M), p-GSK3β (Ser9) (abcam, ab75745), cyclin D1 (Cell Signaling Technology, #2922), pro-caspase-3/cleaved caspase-3 (Cell Signaling Technology, #9662), pro-PARP/cleaved PARP (Cell Signaling Technology, #9532), E-cadherin (Cell Signaling Technology, #14472), Vimentin (Cell Signaling Technology, #5741), β-catenin (Cell Signaling Technology, #8480), Ki-67 (Abclonal, A2094). The secondary antibodies were purchased from Proteintech (Rosemont, IL, USA). OMT was purchased from Aladdin regents (A111285). Taxol was obtained from Aladdin regents (P106869).

### Doubling Time Calculation

A498 and SW839 were seeded at concentrations of 4 × 10^4^ cells per well. After 12 h, cells were treated with 4 mg/ml or 8 mg/ml OMT. After incubated for 24, 48, or 72 h, the cell number was counted by trypan blue staining assay. The doubling time (DT) for each cell line was determined as following: DT (hours) = 0.693(t - t_0_)/ln(Nt/N_0_), t_0_ is the time at which exponential growth began, t is time in hours, Nt is the cell number at time t, and N_0_ is the initial cell number.

### Cell Viability Assay

Cell viability was determined using the Cell Counting Kit-8 (CCK-8) assay. Renal cancer cells were seeded at 4 × 10^3^ cells/well in 96-well plates. After 12 h, the medium was replaced with fresh medium containing indicated concentrations of OMT and incubated for different time points. After incubation, CCK-8 solution was added to each well and incubated at 37°C for an additional 1 h., cells viability was measured as optical density (OD) value of absorbance at wavelength of 450 nm.

### Western Blot

Renal cancer cells with different treatment were collected and lysed with lysis buffer (Beyotime, China). The protein concentrations were assessed *via* Enhanced BCA Protein Assay Kit (Beyotime). Protein (15–30 μg) were separated by electrophoresis in 8%–15% sodium dodecyl sulfate-polyacrylamide minigel (SDS-PAGE), and transferred onto a PVDF membrane. Then membranes were blocked with Tris-buffered saline with tween-20 (TBST) with 5% bovine serum albumin (BSA), and incubated with specific primary antibodies and corresponding secondary antibodies. The protein bands were detected by enhanced chemiluminescence (ECL).

### Cell Cycle Analysis

At 48 h after treatment, cells were collected and washed with PBS, fixed in chilled methanol, and kept at 4°C for at least 2 h. Cells were then collected, resuspended in 500-μl staining buffer, stained with 25 μl propidium iodide (PI) and incubated with 10 μl ribonuclease (RNase). Next, cells were incubated at 37°C for 30 min. Cell cycle analysis was accessed through flow cytometer.

### Apoptosis Assay

Cells were plated in six-well plates and cultured overnight. After treatment, cells were trypsin-released and resuspended with 195 μl binding buffer, adding 5 μl AnnexinV-FITC and 10 μl Propidium iodide. After incubation in dark for 30 min, apoptotic cells were measured by flow cytometer and quantitated.

### Wound Healing Assay

Wound healing assay was employed to detect cell migration ability. Cells were seeded in six-well plates and cultured to confluent state. The medium was replaced with serum-free medium and treated with 1 μg/ml mitomycin C for 1 h. And then cells were treated with OMT or transfected with β-catenin overexpression plasmid for 48 h. Cell monolayers were wounded with a 200 μl pipette tip and washed with serum-free medium to remove detached cells. The wound gap was observed and photographed by inverted phase contrast microscope (OLYMPUS) (at 100× magnification), and the distance of the wound gap was measured. After incubation in serum-free medium for 48 h, the wound gap was photographed again.

### Transwell

Cell invasion ability was detected using 24-well chemotaxis chambers (Corning, CA, USA, Cat: 3422). The upper side of the membrane in the transwell chamber was coated with a mixture of Matrigel (BD, Cat: 356234) and serum-free medium (1:3), and air-dried at 37°C for 2 h. 2 × 10^4^ cells in 200 μl serum-free medium were placed into the upper chambers. The lower chambers were filled with 800 μl medium containing 20% fetal bovine serum (FBS). The cells were treated with OMT or transfected with β-catenin overexpression plasmid for 48 h. The membrane was fixed in 4% paraformaldehyde for 15 min at room temperature, stained with crystal violet for 5 min, and washed with distilled water. The invasiveness of cells was defined by the mean number of cells in five randomly selected microscopic fields (at 200× magnification).

### Immunofluorescence

The cells grown on chamber slides were fixed for 15 min with 4% paraformaldehyde. The samples were permeabilized with 0.1% tritonX-100 for 30 min. Then, the samples were blocked with goat serum for 15 min. Primary antibody against β-catenin diluted in PBS (1:50) were added to the samples and incubated overnight at 4°C. After PBS washing, cells were incubated with Cy3-labeled secondary antibody (1:200, Beyotime, China) diluted in PBS for 1 h at room temperature. The nuclei were stained with DAPI. After three additional 5-minutes washing, samples were sealed by antifade reagent and visualized with an OLUMPUS fluorescence microscope (at 400× magnification).

### Transient Transfection of Renal Cancer Cells

A498 and SW839 cells were transfected by β-catenin overexpression plasmid encapsulated with Lipofectamine 2000 (Invitrogen, Cat:11668-019) according to its transfection protocol for overexpressing β-catenin, or transfected by vector plasmid as the negative control. After 48 h of transfection, a series of assays were performed as described accordingly.

### Drug-Sensitivity Assay

Renal cancer cells were seeded at 4 × 10^3^ cells/well in 96-well plates. After incubation for 12 h, the medium was changed to fresh medium containing various concentrations (0.01, 0.1, 1, 10, 100 μM) of taxol in DMSO. After cells were incubated for 48 h, the viability of cells was measured by CCK-8 assay. The drug concentrations required to cause 50% cell growth inhibition (IC50) were determined by interpolation from the dose-response curves.

### Animal Model and Tissue Processing

Animal experiments were executed according to The Guideline for the Care and Use of Laboratory Animals, and were approved by the Ethics Committee of First Affiliated Hospital of Harbin Medical University of China. Balb/c nude mice aged 4 weeks were used in all studies. The animals were divided randomly into four groups with five mice in each group. A498 or SW839 cells (1 × 10^6^) were injected subcutaneously into the right flank of each mouse. Seven days after injection, the first and third groups of mice were as control, the second and fourth received intraperitoneal injection of OMT (50 mg/kg) treatment every 3 days for six times. The tumor size was measured every 3 days, and the tumor volume was calculated as V=(width^2^×length)/2. The experiment was terminated 28 days after tumor cell inoculation, and mice were sacrificed. The tumors from each mouse were excised and photographed. Part of each tumor was lysed for protein expression analysis through Western blot, and part was sliced and fixed in formalin and embedded in paraffin for immunohistochemical staining.

### Hematoxylin and Eosin Staining

The tissue sections were heated at 60°C for 30 min to perform deparaffinization followed by washing in xylene and rehydration through a graded series of ethanol (100%, 95%, 85%, and 75%) and washed in distilled water. The sections were stained with hematoxylin, differentiated with 1% hydrochloric acid alcohol, and stained with eosin. After subjected to an increasing ethanol series and incubated in xylene, the sections were mounted with coverslip and examined under an OLUMPUS microscope (at 200× magnification).

### TUNEL Staining

In situ cell death detection kit, POD (Roche, Cat: 11684817910) was used for TUNEL staining. The sections were treated for deparaffinage in xylene, rehydrated in ethanol, rinsed in distilled water. They were permeabilized with 0.1% Triton X–100 (diluted in 0.1% citrate buffer) for 8 min at room temperature. The sections were washed with PBS three times for 5 min, then immersed in 3% hydrogen peroxide for 10 min to block endogenous peroxidase activity. The sections were washed with PBS and incubated with TUNEL solution (enzyme solution: label solution = 1: 9) for 60 min at 37°C in dark. After washed with PBS, the sections were incubated with converter-POD solution at 37°C for 30 min. Tissues were washed again with PBS. TUNEL positive cells were stained with diaminobenzidine (DAB), and the slides were counterstained with hematoxylin. Stained slides were differentiated with 1% hydrochloric acid alcohol. Slides were mounted with coverslip after dehydration in ethanol and incubation in xylene. Sections were photographed by an OLUMPUS microscope (at 400× magnification).

### Immunohistochemistry Staining

The slides were heated at 60°C for 2 h, followed by paraffin removal with xylene and subsequent rehydration with ethanol. Antigen retrieval was performed in a chamber containing citrate buffer for 10 min maintaining at a subboiling temperature. Samples were immersed in 3% hydrogen peroxide and then blocked with goat serum for 15 min at room temperature. The primary antibody of Ki-67 (Bioss, 1:100) was added to each slide and incubated overnight at 4°C. HRP-labeled secondary antibody was then applied to incubate for 60 min. The sections were incubated with DAB and counterstained with hematoxylin. Stained slides were differentiated with 1% hydrochloric acid alcohol. After subjected to an increasing ethanol series and incubated in xylene, slides were confined with coverslip and visualized (at 400× magnification).

### Statistical Analysis

Statistical analysis was employed with two-tailed unpaired t-test, one-way ANOVA or two-way ANOVA as indicated in figure legends. Data were represented as mean ± SD deviation. P value was indicated by asterisks in the figures: *P < 0.05, **P < 0.01, ***P < 0.001, ****P < 0.0001. *P* < 0.05 was considered a statistically significant difference.

## Results

### OMT Inhibited the Proliferation of Renal Cancer Cells

**Figure 1:**
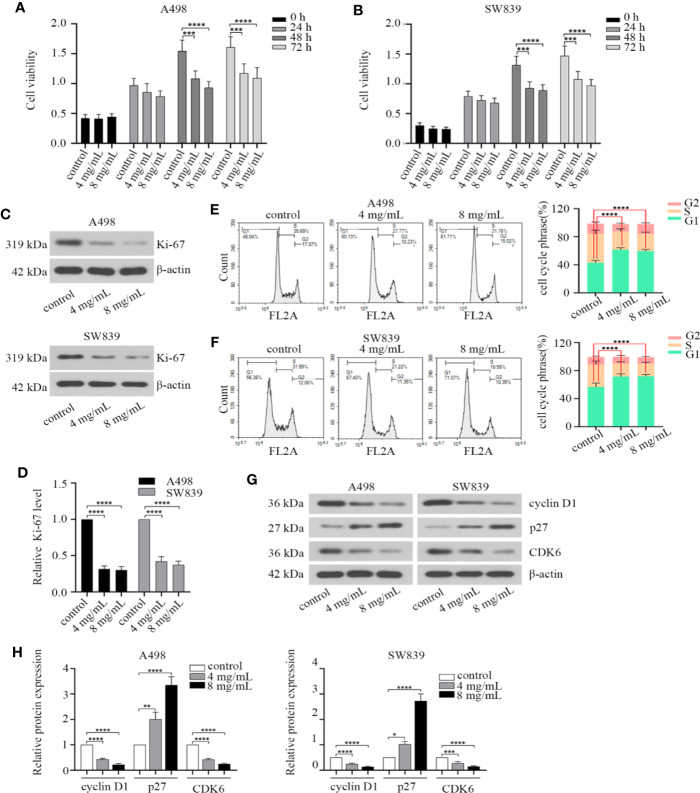
Oxymatrine inhibited the proliferation of renal cancer cells. **(A, B)** Human A498 and SW839 cells were treated with oxymatrine (4 mg/ml, 8 mg/ml) for 24, 48 and 72 h, cell viability was detected by Cell Counting Kit-8 (CCK-8) assay. The results were the mean ± SD of three experiments with three determinations of per sample, and two-way ANOVA was shown (***P < 0.001, ****P < 0.0001). A498 and SW839 cells were preincubated with oxymatrine, 48 h after treatment, **(C)** Western blot was employed to examine Ki-67 protein expression. **(D)** The quantitative analysis of Ki-67 protein was performed (****P < 0.0001). **(E, F)** Cell cycle was analyzed by flow cytometry and quantitative analysis of cell cycle phrase was performed, the data was presented as mean ± SD (n=3) of three independent experiments (****P < 0.0001). **(G)** The expression level of cell cycle-associated protein markers was detected by Western blot; **(H)** The quantitative analysis of the proteins in G with statistic significance calculated from one-way ANOVA (*P < 0.05, **P < 0. 01, ***P < 0.001, ****P < 0.0001).

We first treated A498 and SW839 cells with different concentrations of OMT (0, 0.5, 1, 2, 4, 8, 10 mg/ml) for 48 h to test cell viability. The two lowest concentrations (4 mg/ml, 8 mg/ml) that inhibited cell activity by more than 20% were chosen to treat A498 and SW839 cells for subsequent studies. To characterize the effect of OMT on renal cancer cell proliferation, doubling time and cell viability were determined in A498 and SW839 cells. RCC cells treated with OMT had the longer doubling time compared with control cells (**Supplementary Figure 1**). Treatment with OMT for 48 h and 72 h significantly inhibited the viability of RCC cells (**Figures 1A, B**). Ki-67 is present during all active phases of the cell cycle (G1, S, G2 and mitosis), and absent in resting cells (G0 phase), which is a biomarker for reflects the tumor cell proliferation rate (Gerdes et al., 1984). Based on this, we detected the effect of OMT on Ki-67 protein expression. The cells treated with OMT displayed a remarkable decrease in Ki-67 expression (**Figures 1C, D**). Given that cell cycle arrest leads to decreased cell proliferation, we next analyzed the effect of OMT on this biological function in renal cancer cells. The results indicated that much higher percentages of RCC cells were rested in G1 phase upon OMT treatment (**Figures 1E, F**). Moreover, the expression of some key regulators involved in G1 phase was prominently changed after cells were treated with OMT. The level of cyclin D1 and CDK6 protein was reduced and p27 was elevated (**Figures 1G, H**). These results together suggested that OMT restrained renal cancer cells proliferation by inducing cell cycle arrest.

### OMT Induced Apoptosis of Renal Cancer Cells

**Figure 2:**
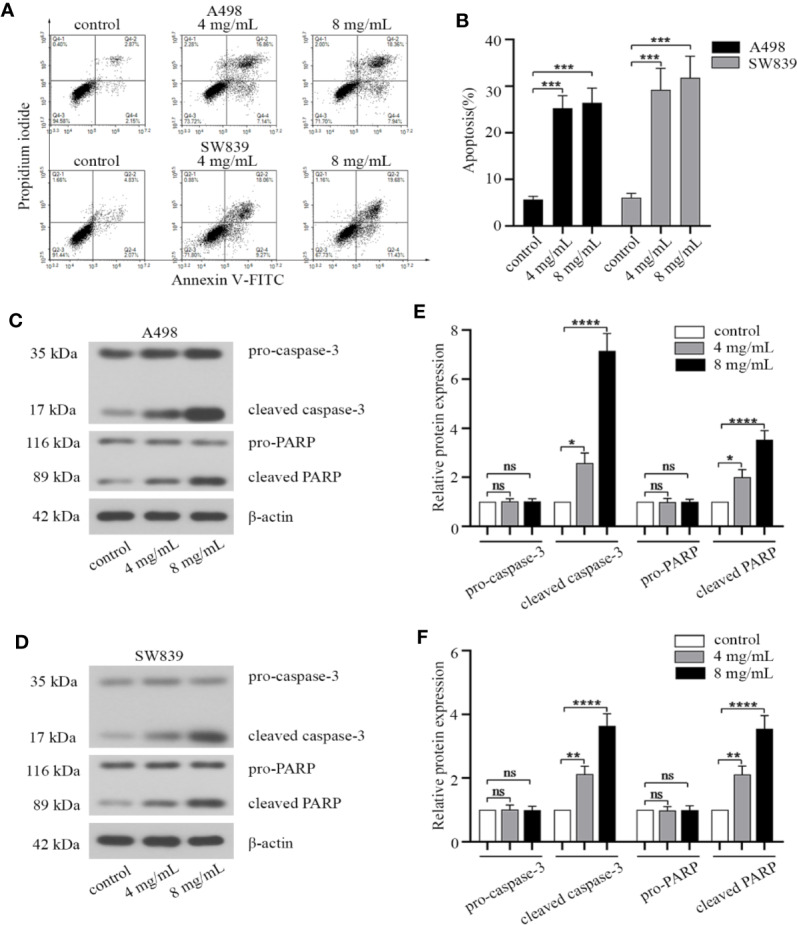
Oxymatrine induced apoptosis of renal cancer cells. A498 and SW839 cells were preincubated with oxymatrine (4 mg/ml, 8 mg/ml) for 48 h. **(A)** The cells were double stained with Annexin V-FITC/PI and analyzed by FACS, and the experiment was repeated three times. **(B)** The percentage of apoptotic cells was calculated as mean ± SD with one-way ANOVA (***P < 0.001). **(C, D)** The protein levels of apoptosis-related markers in A498 and SW839 cells were determined by Western blot. **(E, F)** The quantitative analysis of pro-caspase-3, cleaved caspase-3, pro-PARP, cleaved PARP proteins was performed (*P < 0.05, **P < 0. 01, ****P < 0.0001).

Since apoptotic pathway was considered as a potential therapeutic target for cancer treatment (Baig et al., 2016). We investigated the effect of OMT on apoptosis by FACS, in order to identify whether the inhibition of cell proliferation caused by OMT is associated with the increased activation of the apoptotic pathway. By Annexin V-FITC/PI staining, a remarkable increase in cell apoptosis was observed in A498 and SW839 cells treated with OMT (**Figures 2A, B**). We subsequently examined the effect of OMT on the expression of key apoptotic proteins in A498 and SW839 cells by Western blot. As shown **Figures 2C–F**, the level of cleaved caspase-3 and cleaved PARP was increased in OMT-treated A498 and SW839 cells. These data proved a role for OMT in inducing apoptosis of renal cancer cells.

### OMT Suppressed Migration and Invasion of Renal Cancer Cells

**Figure 3:**
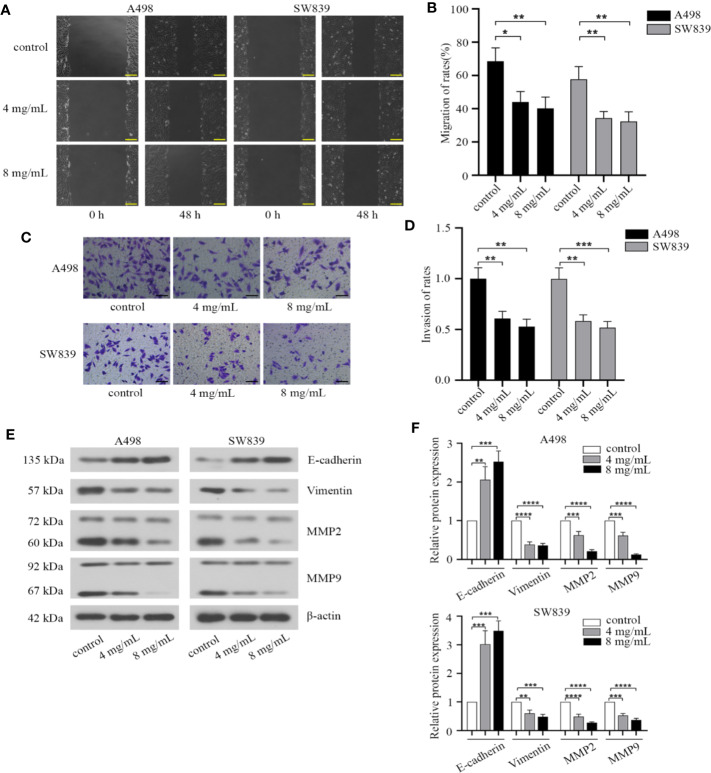
Oxymatrine suppressed migration and invasion of renal cancer cells. **(A)** Wound-healing assay in A498 and SW839 cells treated with oxymatrine (4 mg/ml, 8 mg/ml) for 48 h. Scale bar, 200 μm. **(B)** Quantitative analysis of migration rates. **(C)** A498 and SW839 cells were detected by transwell with matrigel after oxymatrine treated for 48 h. Scale bar, 100 μm. **(D)** Quantitative analysis of invasion rates. **(E)** The expression of epithelial to mesenchymal transition(EMT) and MMP-associated markers in A498 and SW839 cells following oxymatrine treated for 48 h. **(F)** Quantitative analysis of proteins in E was presented. The quantitative analysis for the migration distance and invaded number of cells in B and D was presented as mean ± SD calculated based on three separate experiments with one-way ANOVA (*P < 0.05, **P < 0. 01, ***P < 0.001, ****P < 0.0001).

We then investigated the effect of OMT on migration and invasion in renal cancer cells. By employing a wound healing assay, we observed that the wounding space between cell layers after scratching was less occupied after 48 h in cells treated with OMT (4 mg/ml, 8 mg/ml) compared with control cells (**Figures 3A, B**). Based on transwell assay, treatment with OMT in A498 and SW839 cells dramatically decreased the number of cells penetrated (**Figures 3C, D**). Furthermore, we determined the expression of matrix metalloproteinases involved in the cell migration, as well as epithelial to mesenchymal transition (EMT) markers involved in cell invasion. As shown in Western blot analysis, exposure to OMT of A498 and SW839 cells for 48 h markedly reduced levels of Vimentin, MMP2, MMP9 and improved E-cadherin level (**Figures 3E, F**). Taken together, our results demonstrated that OMT inhibited migration and invasion in renal cancer cells.

### OMT Affected the Nuclear Localization of β-Catenin in Renal Cancer Cells

**Figure 4:**
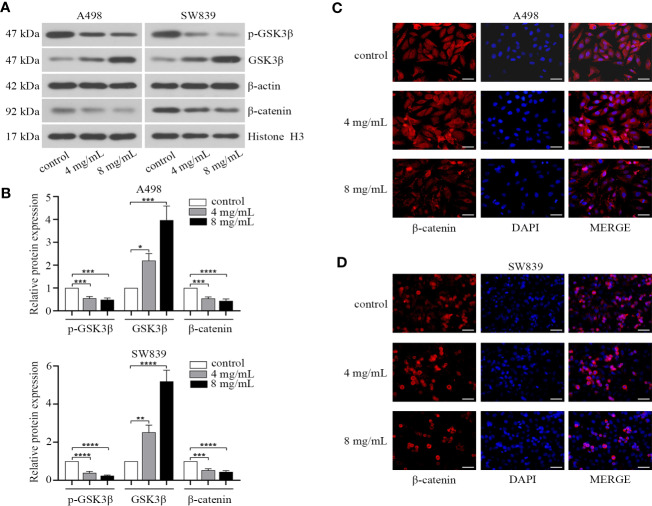
Oxymatrine affected the nuclear localization of β-catenin in renal cancer cells. A498 and SW839 cells were treated with oxymatrine (4 mg/ml, 8 mg/ml) for 48 h. **(A)** The expression of GSK3β, p-GSK3β (Ser9) in the whole cell lysate and β-catenin in the nucleus tested by Western blot. **(B)** The analysis of relative protein expression in A (*P < 0.05, **P < 0. 01, ***P < 0.001, ****P < 0.0001). **(C, D)** Immunofluorescence analysis was performed to observe the expression and localization of β-catenin. Scale bar, 50 μm.

As EMT progression could be controlled by Wnt/β-catenin pathway in human cancer (Howe et al., 2003), and several pieces of data have also demonstrated the role of β-catenin in renal cancer (Thievessen et al., 2003; Bruder et al., 2007; Krabbe et al., 2014). We hypothesized that OMT might influence EMT progression of renal cancer through this signaling pathway. Thus, we identified the effect of OMT on the expression of β-catenin and key proteins involved in Wnt/β-catenin pathway in renal cancer cells. The results showed that OMT treatment (4 mg/ml, 8 mg/ml) not only increased GSK3β at protein level, but also enhanced its kinase activity (as evidenced by the decrease in phosphorylation of GSK3β at Ser9), which led to reduced nuclear accumulation of β-catenin (**Figures 4A, B**). To confirm the effect of OMT on the localization of β-catenin in renal cancer cells, immunofluorescence analysis was performed and further demonstrated the decreased β-catenin translocation to the nucleus (**Figures 4C, D**). These results elucidated that OMT restrained the expression and translocation of β-catenin in renal cancer cells.

### OMT Suppressed the Progression of Renal Cancer by Inhibiting β-Catenin Expression

**Figure 5:**
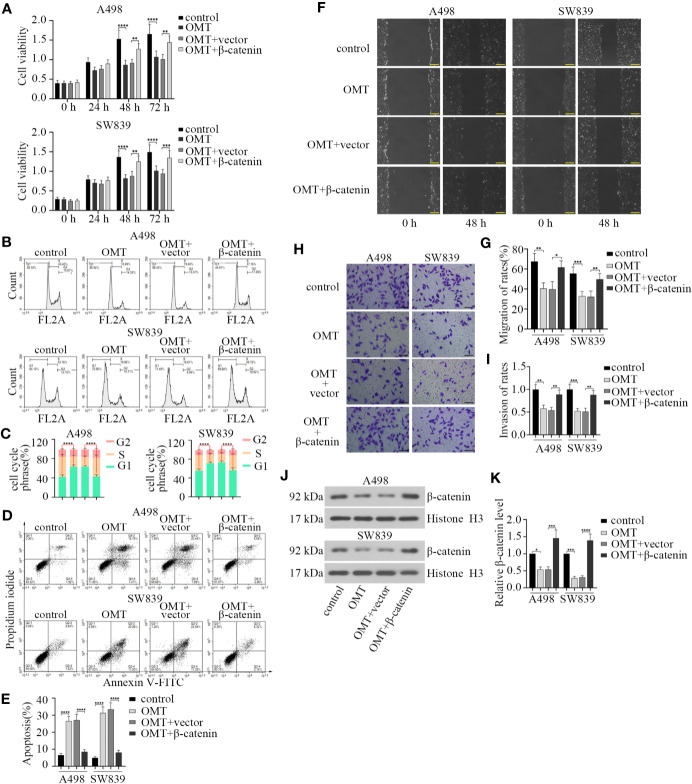
Oxymatrine suppressed the progression of renal cancer by inhibiting β-catenin expression. A498 and SW839 cells were treated with oxymatrine following transfected with β-catenin overexpression plasmid, and **(A)** After 24, 48, and 72 h of treatment, cell viability was determined by CCK-8 assay. **(B)** Flow cytometry analysis was performed to detect cell cycle after 48 h of treatment. **(C)** Quantitative analysis of cell cycle phrase. **(D)** The apoptosis cells were analyzed by FACS. **(E)** Quantification of apoptosis (%). **(F)** Cell migration was tested by wound-healing assay after cells were treated for 48 h. **(G)** The migration of rates was calculated. **(H)** Cell invasion was determined by transwell assay. **(I)** The rates of invasion cells were calculated. **(J)** The protein levels of β-catenin in the nucleus detected by Western blot. **(K)** The quantitative analysis of β-catenin protein level. Data in panel **A, C, E, G**, **I** were shown as the mean ± SD from three separate experiments, with two-way ANOVA for A, C and one-way ANOVA for E, G, I, and K (*P < 0.05, **P < 0. 01, ***P < 0.001, ****P < 0.0001).

As noted above, treatment with OMT influenced expression and nuclear localization of β-catenin. We aimed to verify the involvement of β-catenin in OMT-suppressed renal cancer progression. We first transfected β-catenin overexpression plasmid into A498 and SW839 cells, respectively. After treatment with OMT (8 mg/ml) for 24, 48, and 72 h, cell proliferation capacity was tested by CCK-8 assay. As shown in **Figure 5A**, OMT prominently inhibited cell viability, while this was restored by transfection with β-catenin overexpression plasmid. To confirm OMT's repression of cell proliferation caused by cell cycle arrest, we employed cell cycle analysis by flow cytometer. Results revealed that overexpression of β-catenin abolished the arrests at G1 phase of the cell cycle caused by OMT (**Figures 5B, C**). Additionally, β-catenin overexpression reversed OMT-induced apoptosis in renal cancer cells (**Figures 5D, E**). Furthermore, wound healing assay was performed and exhibited overexpressed β-catenin increased the migration capacity inhibited by OMT (**Figures 5F, G**). Similarly, OMT-restrained invasion capacity was abolished by the overexpression of β-catenin (**Figures 5H, I**). We also determined the level of β-catenin in the nucleus through Western blot. OMT decreased the level of nuclear β-catenin, while it was reversed by β-catenin overexpression (**Figures 5J, K**). The above results illustrated that OMT suppressed renal cancer progression by inhibiting β-catenin expression.

### OMT Facilitated the Sensitivity of Renal Cancer Cells to Chemotherapy

**Figure 6:**
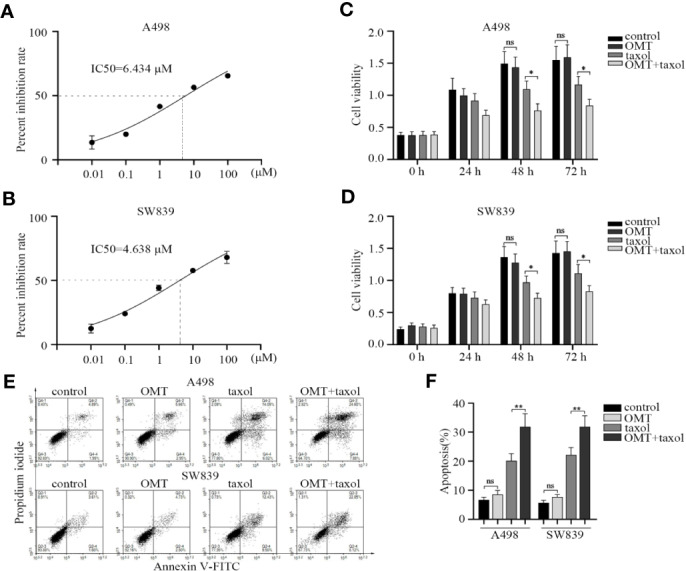
Oxymatrine facilitated the sensitivity of renal cancer cells to chemotherapy. **(A, B)** A498 and SW839 cells were treated with taxol at the indicated doses and its IC50 value was calculated accordingly. A498 and SW839 cells were treated with oxymatrine or taxol alone or their combination, **(C, D)** Cell Counting Kit-8 (CCK-8) assay was employed after 24, 48, and 72 h to detect cell viability. **(E)** After 48-h treatment, the apoptosis was determined by FACS. **(F)** The percentage of apoptotic cells was calculated. Data in C, D and F were shown as mean ± SD of three experiments (*P < 0.05, **P < 0. 01).

Considering the contribution of OMT in enhancing the sensitivity of cancer cells to chemotherapy drugs (Li et al., 2014; Liu et al., 2016), we reasoned that OMT may be involved in drug sensitivity of renal cancer cell. First, we identified the effect of taxol treatment on cell viability inhibition. The IC50 value of taxol in A498 and SW839 cells was 6.434 μM (0.0055 mg/ml) and 4.638 μM (0.004 mg/ml), respectively (**Figures 6A, B**). Next, we chose the maximum concentration of OMT (1 mg/ml) that did not affect cell viability to cotreated cells with taxol (one-third of the IC50 value) for 24, 48, and 72 h. CCK-8 assay was employed to further evaluate the synergistic inhibitory effect of taxol and OMT. The combined treatment with OMT and taxol markedly facilitated taxol-modulated inhibition of cell viability as compared with cells treated with taxol alone (**Figures 6C, D**). Meanwhile, treatment with taxol alone induced 20.11% and 21.93% apoptotic cells in A498 and SW839 cells respectively. However, the addition of OMT significantly increased taxol-induced apoptosis, leading to a 32.48% and 30.97% induction of apoptosis in A498 and SW839 cells respectively at 48 h after treatment (**Figures 6E, F**). These results proved that the combinational use of OMT and taxol resulted in improved sensitivity in renal cancer cells to chemotherapy drug.

### OMT Suppressed Tumor Progression in Mouse Models With Renal Cancer Cells Xenografts

**Figure 7:**
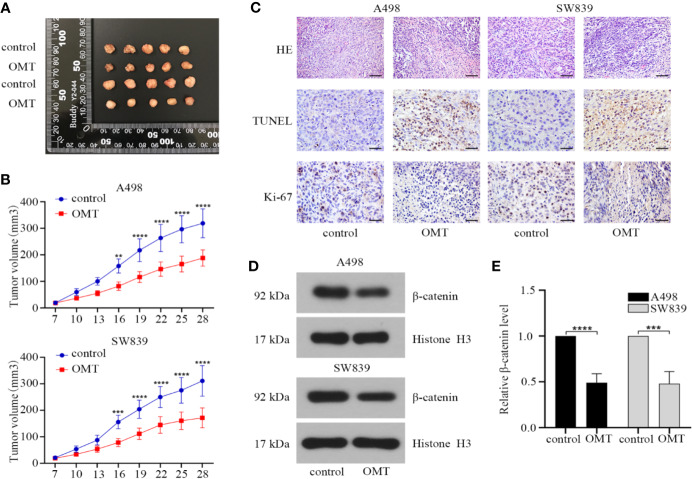
Oxymatrine suppressed tumor progression in mouse models with renal cancer cells xenografts. **(A)** The xenografts of A498 and SW839 cells were harvested at 28 days after transplantation, and the morphology was photographed. Upper panels: the xenografts of A498 cells. Lower panels: the xenografts of SW839 cells**. (B)** Tumor diameters were measured at a regular interval of 3 days for up to 28 days and the tumor volume was calculated. Error bars, mean ± SD. **P < 0.01, ***P < 0.001, ****P < 0.0001 (two-way ANOVA). **(C)** hematoxylin and eosin (HE) staining (scale bar, 100 μm), TUNEL staining (scale bar, 50 μm) and immunohistochemistry staining (scale bar, 50 μm) of Ki-67 from tumor tissue sections. **(D)** The protein expression of β-catenin in the nucleus of tumor tissues determined by Western blot. **(E)** Relative β-catenin protein level in D was calculated.

Based on the results from *in vitro* studies, we further validated effect of OMT on tumorigenesis *in vivo* using renal cancer cell-derived xenografts. A498 or SW839 cells (1 × 10^6^) were subcutaneously injected into BALB/c nude mice that were divided into 4 groups (n = 5 for each group). During 21 days of administration with OMT, tumor volume was monitored and protein expressions in tumor tissues were analyzed after the mice were sacrificed. As shown in **Figures 7A, B**, treatment with OMT effectively inhibited *in vivo* tumor growth. Furthermore, hematoxylin and eosin (HE) staining showed that the tumor cells of control mice were in irregular shape and with abundant cytoplasm and large and deformed nuclei. However, in the tumor cells of the mice treated with OMT, the nuclei were smaller and in more regular shape. TUNEL staining and immunohistochemical staining of Ki-67 respectively illustrated OMT promoted apoptosis and suppressed proliferation *in vivo* (**Figure 7C**). Moreover, Western blot of tissue lysates of the xenograft tumors revealed that treatment with OMT in the mice decreased the level of β-catenin in the nucleus (**Figures 7D, E**). All these results confirmed that OMT inhibited the growth of human renal cancer cell xenograft.

## Discussion

In recent years, the incidence of RCC continues to increase worldwide. Although patients that present with early stage of RCC can be treated with surgery, the 5-year survival rate for patients with advanced stage is dramatically reduced because of the recurrence and metastasis after tumor resection. Most RCC patients who received cytotoxic or targeted drug therapy develop resistance at some point during treatment. Therefore, identification of new and highly effective drugs and therapeutic targets is considered to improve current therapies for RCC. Here, we demonstrated that OMT had marked antitumor effect accompanied by decreased proliferation, migration and invasion as well as enhanced apoptosis and drug sensitivity. Notably, we further verified that OMT played its antineoplastic role by suppressing β-catenin in RCC. Our study provided new insights into how OMT serves as an effective drug in RCC treatment and associated molecular mechanisms.

It has been demonstrated that aberrant signal transduction of Wnt/β-catenin is correlated with an aggressive phenotype in RCC (SA et al., 2014; Xu et al., 2016). Moreover, inhibition of β-catenin, the key regulator of Wnt pathway, can restrain cancer cell growth, trigger apoptosis activity and prevent chemoresistance (Behari et al., 2007; Yasui et al., 2007; Wickstrom et al., 2015). In this study, treatment with OMT in RCC cells affected the expression and localization of β-catenin. Overexpression of β-catenin reversed the suppression of proliferation, migration and invasion as well as induction of apoptosis caused by OMT in subsequent rescue experiments. All these findings proved that OMT targeted β-catenin, so it is rational to deduce that OMT restrained the progression of RRC *via* inhibiting nuclear translocation of β-catenin. Similarly, Zhang et al. demonstrated that OMT caused a decrease in the side population (cancer stem-like cells) *via* Wnt/β-catenin signaling pathway (Zhang et al., 2011). This finding was concordant to our research, the repressive effect of OMT on cancer cells was caused by inhibiting the expression of β-catenin.

Given that aberrant cell cycle is a hallmark of cancer and Cyclin D1 is a key regulator in G1-S transition of cell cycle (Hanahan and Weinberg, 2011; Otto and Sicinski, 2017). Overwhelming publications have described that the expression of cyclin D1 frequently elevates in RCC (Hedberg et al., 1999; Bindra et al., 2002). It has reported that cyclin D1 could be transcriptionally activated by β-catenin in the nucleus (Botrugno et al., 2004; Maretzky et al., 2005). Here, we found that OMT caused cell cycle arrest at G1 phrase accompanied with cyclin D1 downregulation, and exogenous overexpression of β-catenin rescued the cell cycle arrest at G1 phrase in OMT-treated cells (**Figures 1** and **5**). There is a possibility that OMT inhibited β-catenin nuclear translocation and β-catenin-mediated transcriptional regulation of cyclin D1 in cell cycle of RCC.

In addition to the effect on cell cycle, we revealed that OMT restrained migration and invasion in RCC cells *via* repressing β-catenin. Meanwhile, reduced E-cadherin expression was observed in Western blot after treated with OMT. Several studies have shown that the disruption of E-cadherin/β-catenin complex results in tumor metastasis in a series of epithelial malignancies (Kandouz et al., 2010; Chen et al., 2016). Moreover, EMT progression is under the control of Wnt/β-catenin pathway. OMT may interact with and stabilize E-cadherin/β-catenin complex, thereby inhibiting redistribution of β-catenin to the nucleus and its transcriptional activity, further preventing EMT progression. To test this hypothesis, more detailed investigations need to be performed in future study.

Recently, several studies suggested that OMT exerts synergistic antitumor effects with oxaliplatin in colon cancer cells (Liu et al., 2016) and sensitizes cisplatin-resistant cervical cancer cells to the cytotoxic effects of cisplatin (Li et al., 2014). Thus, OMT has the potential to serve as a chemosensitizer for RCC treatment *via* amplifying the effectiveness of chemotherapy drugs. Taxol is a chemotherapeutics used to treat various types of cancers. It arrests the cell cycle in G0/G1 and G2/M phases *via* binding to the β-subunit of tubulin and stabilizing microtubules, which ultimately leads to apoptosis (Long, 1994). Although taxol has been commonly used for the treatment of cancer patients, intrinsic and acquired resistance to it becomes a notable clinical problem currently (Saloustros et al., 2008). Considering these, we employed CCK-8 and apoptosis assay to detect whether OMT can enhance the efficacy of taxol in RCC. As shown in **Figure 6**, the combination with OMT and taxol facilitated the taxol-modulated inhibition of cell viability and induction of apoptosis. The finding above provided a novel therapeutic strategy in RCC.

A large body of literature has verified that approximately 80% of RCC is caused by the biallelic inactivation of the VHL gene (Kaelin, 2002; Barry and Krek, 2004). The VHL gene product (pVHL) is a part of ubiquitin ligase complex that targets β-catenin for degradation (Linehan et al., 2009). It has also been demonstrated by a systematic study of RCC clinical samples that β-catenin level is increased in the case of VHL deficiency (Lian et al., 2012). In VHL-deficient RCC cells (such as A498), the ratio of activated β-catenin in the nucleus is higher than that in VHL-positive cells (Dere et al., 2015). Considering these, we chose A498 (VHL-negative cell line) and SW839 (VHL mutant cell line) to study the effects of OMT on renal cancer by modulating β-catenin.

In summary, our study reported OMT-induced tumor suppression in RCC progression. Further, the realization of such effect was dependent on its inhibition on β-catenin. Thus, this study provided a preclinical basis that OMT assisted suppression of RCC development, and it could be a promising therapeutic approach in RCC treatment.

## Data Availability Statement

The datasets generated for this study are available on request to the corresponding author.

## Ethics Statement

The animal study was reviewed and approved by the Ethics Committee of First Affiliated Hospital of Harbin Medical University of China.

## Author Contributions

RA conceived the study, participated in the designing of the study, and coordinated the study. YJ prepared the manuscript. JL, YadL, YanL, GG, and SY performed the experiments. YJ analyzed the data. GG collected the fund. All authors have read and approved the final manuscript.

## Funding

This study was supported by a grant from the Post-doctoral Foundation of Heilongjiang Province (No. LBH-Z16243).

## Conflict of Interest

The authors declare that the research was conducted in the absence of any commercial or financial relationships that could be construed as a potential conflict of interest.

## Supplementary Material

Supplementary Figure 1 Effect of Oxymatrine on doubling time of renal cancer cells. **(A**, **B)** A498 and SW839 cells were treated with oxymatrine at the indicated doses. Cell numbers were counted and doubling time was calculated. Comparisons between different groups were made using one-way ANOVA.

## Abbreviations

CCK-8, Cell Counting Kit-8; DAB, diaminobenzidine; EMT, epithelial to mesenchymal transition; HE staining, hematoxylin and eosin staining; IC50, the half maximal inhibitory concentration; OMT, oxymatrine; RCC, renal cell carcinoma.
