# Supplementary figures and images for: Oxymatrine Inhibits Renal Cell Carcinoma Progression by Suppressing β-Catenin Expression
Source: Front Pharmacol. 2020 Jun 5;11:808. doi: 10.3389/fphar.2020.00808 (PMC7289957; doi:10.3389/fphar.2020.00808)

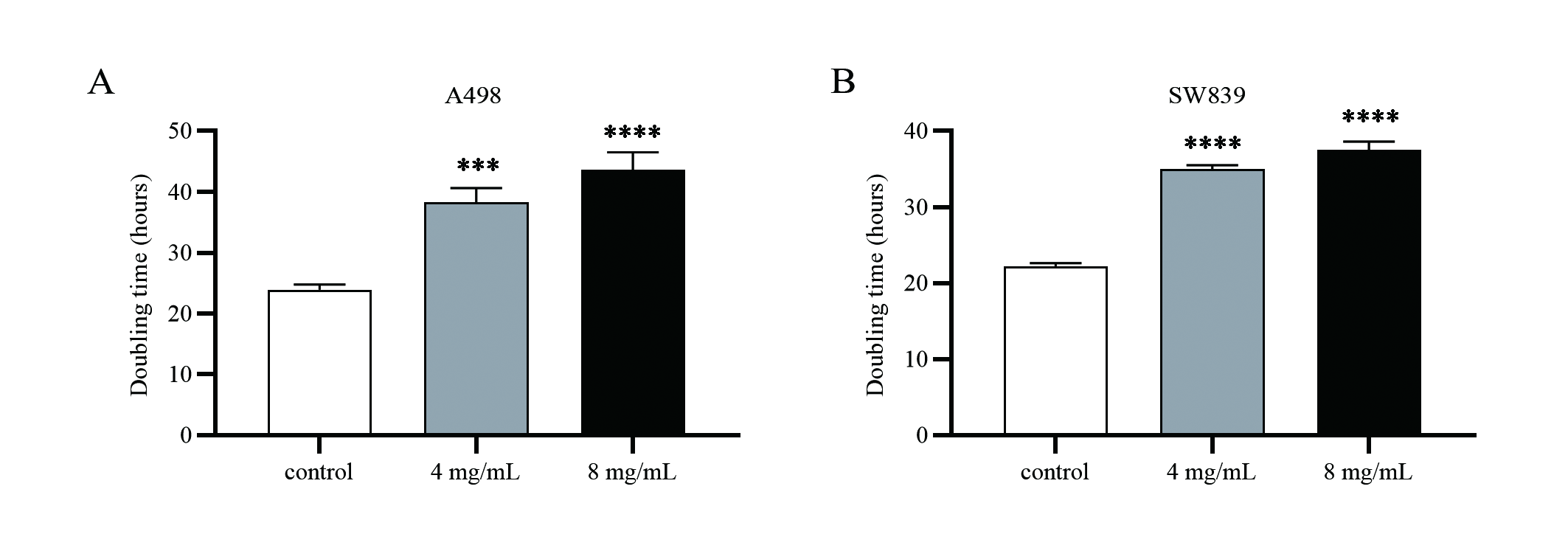

Supplement: Supplementary Figure 1 — Effect of Oxymatrine on doubling time of renal cancer cells. (A, B) A498 and SW839 cells were treated with oxymatrine at the indicated doses. Cell numbers were counted and doubling time was calculated. Comparisons between different groups were made using one-way ANOVA. [file Image_1.tif]
